# Linking hair cortisol and life stress: The role of stress reactivity and habituation

**DOI:** 10.1016/j.psyneuen.2025.107715

**Published:** 2025-12-02

**Authors:** Jari Planert, Tobias Stalder, Katharina Huthsteiner, George M. Slavich, Tim Klucken, Johannes B. Finke

**Affiliations:** a Clinical Psychology and Psychotherapy, Department of Psychology, University of Siegen, Siegen, Germany; b Department of Psychiatry and Biobehavioral Sciences, University of California, Los Angeles, CA, USA

**Keywords:** Hair cortisol, Chronic stress, Acute stress, Stress habituation, Trier social stress test, Early-life adversity

## Abstract

**Background:**

Hair cortisol concentration (HCC) has emerged as a biomarker for long-term cortisol secretion, yet evidence linking HCC to self-reported life stress remains inconsistent. Although individual differences in acute stress reactivity as well as habituation may moderate this association, no research has examined how these processes interact to modulate the HCC-stress link. Moreover, most studies have relied on assessments of recent stressor exposure only, with limited attention to lifetime stressor exposure.

**Method:**

A final sample of 72 healthy individuals (53 women) who provided hair samples and underwent the Trier Social Stress Test three times over consecutive weeks, during which changes in salivary cortisol, cardiovascular parameters, and self-reported stress were assessed. The Stress and Adversity Inventory was administered to assess lifetime stressor exposure.

**Results:**

As hypothesized, preregistered analyses showed that greater lifetime stressor exposure and acute cortisol reactivity were both associated with elevated HCC. No association was found between HCC and stress habituation, and no moderation effects on the relation between HCC and lifetime stressor exposure were observed for reactivity or habituation. Exploratory analyses revealed a consistent link between early-life stressor exposure and HCC, whereas a positive association with adulthood stressors was evident only for individuals with less cortisol reactivity.

**Conclusions:**

The results suggest that HCC reflects not only lifetime stressor exposure but is also influenced by individual differences in cortisol reactivity, highlighting its role as an integrative, yet complex biomarker of chronic stress. In contrast, the lack of an association with habituation indicates limited sensitivity to dynamic adaptation processes occurring over weeks.

## 1. Introduction

Obtaining a stable measure of long-term cortisol secretion has long posed a methodological challenge in stress research, as repeated sampling of urine, blood, or saliva is often impractical or invasive (Hellhammer et al., 2009). Hair cortisol concentration (HCC) has emerged as a promising biomarker of long-term stress, offering a non-invasive and time-efficient alternative to traditional methods (Stalder and Kirschbaum, 2012). Assuming that cortisol is continuously deposited in growing hair, assessment of HCC is thought to enable a retrospective analysis of cumulative systemic cortisol exposure over months. However, the extent to which HCC accurately reflects chronic stress remains a matter of debate. While several studies report elevated HCC under sustained stressor exposure, findings are heterogeneous, and meta-analytic work has emphasized that HCC primarily indexes long-term cortisol accumulation rather than subjective or chronic stress per se (Stalder et al., 2017; Steudte-Schmiedgen et al., 2016). Evidence linking HCC to self-reported questionnaire-based stress assessments has been mixed (Stalder et al., 2017), with several studies demonstrating elevated HCC in high-stress populations (e.g., caregivers, individuals experiencing long-term unemployment; Dettenborn et al., 2010; Stalder et al., 2014; Staufenbiel et al., 2013), particularly if stress is still ongoing in these groups (Stalder et al., 2017). To assess stressor exposure, most studies have used established, yet short, highly condensed questionnaires, which may lead to issues with reduced construct validity, memory bias, etc., calling for an approach that investigates links to HCC based on more robust, behaviorally anchored assessments of stressors occurring over the entire life course (Planert et al., 2023; Shields and Slavich, 2017).

To support a causal relationship between HCC and self-reported stress, reliable associations would have to be established, ideally reflecting a dose-response pattern. In prior research, lifetime stressor exposure has been related to biological markers of stress, including altered diurnal cortisol (Lam et al., 2019) and immunological parameters (Olvera Alvarez et al., 2019). While there are studies that have investigated the link between HCC and, for example, early-life stressors (Karlén et al., 2011) or exposure to specific types of adversity (Staufenbiel et al., 2013), comprehensive research on HCC and life stress remains scarce (Stalder et al., 2017). In the past, several instruments have been devised that aim to comprehensively assess stressor exposure across the life course, such as the Life Events and Difficulties Schedule (Monroe et al., 2007) or the Traumatic Life Events Questionnaire (Kubany et al., 2000). A more recent approach that simultaneously captures both the count and severity of acute as well as chronic stressors is the Stress and Adversity Inventory (STRAIN; Slavich and Shields, 2018).

In addition to enhancing the content validity of long-term stress assessment, other mechanisms that contribute to variation in cortisol deposition in human hair, and thereby heterogeneity in prior findings (Stalder et al., 2017; Steudte-Schmiedgen et al., 2015), should be taken into account. One such factor (closely linked to cortisol secretion) is acute reactivity to psychosocial stressors (Dickerson and Kemeny, 2004), combining elements of social evaluation, performance pressure, and unpredictability (Kirschbaum et al., 1993). Experimental research has revealed substantial individual differences in stress reactivity, which some authors have described as a relatively stable, trait-like response tendency, although this view remains debated, given that stress reactivity also varies across time and context (Boyce and Ellis, 2005; Kudielka et al., 2009) Also, stress reactivity has been associated with the etiology of mental disorders (Heim et al., 2000; McEwen, 2007; Turner et al., 2020), although it remains debated whether altered reactivity constitutes a causal risk factor for or rather a concomitant of psychopathology or trauma (Miller et al., 2007). Despite prior research having provided valuable insights, findings regarding HCC remain inconclusive: Some studies have reported (trend-level) associations between acute cortisol reactivity and HCC (Steudte-Schmiedgen et al., 2015), others have not (Steudte-Schmiedgen et al., 2017). That is, presumably, because the complexity of stress regulation is not fully captured by single-session stress assessments.

Apart from variation in initial stress reactivity, differences in the capacity to adapt to stressors across repeated exposures may also play a critical role in the long-term accumulation of cortisol. Progressive reductions in neuroendocrine reactivity when confronted repeatedly with similar stressors, usually termed ‘habituation’, are well-documented (e.g., Schommer et al., 2003; Wüst et al., 2005). Consistent with the notion of allostatic load—that is, the physiological wear and tear from chronic overactivation of stress response systems due to prolonged exposure and/or inefficient adaptation to repeated bouts of stress (McEwen, 1998a), quick habituation of the stress response is often considered adaptive, whereas weak habituation or even sensitization may indicate difficulties in down-regulation and thus potentially lead to chronic, elevated cortisol secretion (Wüst et al., 2005). Compared to stress reactivity, considerably less research has investigated its habituation, as this poses significant methodological challenges (Quaedflieg et al., 2018; Schommer et al., 2003). For instance, to comprehensively characterize the habituation of stress reactivity, three consecutive stress exposures at fixed intervals are required (Wüst et al., 2005). Taken together, as it is conceivable that HCC does not merely reflect accumulated stress, but possibly also integrates cortisol secretion in response to repeated acute stress, how effectively an individual adapts to repeated exposure may be an important modulatory factor. However, research into the triad of HCC, lifetime stressor exposure, and stress reactivity is missing, specifically regarding the role of habituation.

To address these gaps, the present study examined associations between lifetime stressor exposure, cortisol reactivity and habituation, and HCC. Based on evidence that greater cumulative stressor exposure is related to elevated long-term cortisol secretion (Karlén et al., 2011; Stalder et al., 2017), we hypothesized that lifetime stress would be associated with HCC (H1). Second, given prior research indicating that HCC may integrate both cumulative stressor exposure and acute stress reactivity (Sandner et al., 2020; Steudte-Schmiedgen et al., 2015), we hypothesized (https://osf.io/5br2u) that individuals with greater cortisol reactivity would exhibit greater HCC (H2a). Conversely, based on studies suggesting that efficient habituation indicates adaptive stress regulation (Schommer et al., 2003; Wüst et al., 2005), we hypothesized a negative association between the extent of habituation and HCC (H2b). Finally, building on the notion that individual differences in stress reactivity and habituation might influence the biological embedding of life stress (Gianferante et al., 2014; Lam et al., 2019), we hypothesized that the association between lifetime stressor exposure and HCC would be moderated by stress reactivity (H3a) and stress reactivity habituation (H3b). Additionally, in an exploratory fashion, we tested whether the proposed associations (H3) differ when considering stressor exposure during different developmental periods. To address these questions, healthy individuals provided hair samples for endocrine analyses and underwent a detailed lifetime stressor assessment using the STRAIN (Slavich and Shields, 2018) as well as three subsequent stress inductions via the TSST (Kirschbaum et al., 1993).

## 2. Material and methods

### 2.1. Participants

Eighty-two healthy adults were recruited from the general population via announcements, flyers, and university-wide e-mail distribution lists. Eligibility was determined via a semi-structured telephone screening assessing all exclusion criteria, including self-reported absence of current psychiatric symptoms and diagnoses. Inclusion criteria comprised having hair of at least 2 cm in length at the posterior vertex region (as defined by Huthsteiner et al., 2025) and the absence of any of the following exclusion criteria: current psychotherapy, current psychiatric diagnoses, endocrine disorders, cardiovascular diseases, regular smoking (> 5 cigarettes per day), intake of medication influencing mood, cognition, or endocrine function (e.g., corticosteroids; except hormonal contraceptives). For females, continuous intake of oral contraceptives for at least three months before study entry was required to control for hormonal fluctuations over the four-week study period (Montero-López et al., 2018). As oral contraceptives suppress endogenous estradiol and progesterone secretion, and the associated withdrawal bleeding does not constitute a true menstrual phase, testing was assumed to have occurred under hormonally stable conditions for all female participants (Pletzer and Kerschbaum, 2014; Wiegratz and Kuhl, 2004). After participation in the presession, six participants did not show up for the first TSST session, and an additional four participants dropped out following the first TSST. These individuals were excluded from all analyses as deriving indices of stress reactivity and habituation required data from at least two TSST sessions (final sample for analyses of stress reactivity: *N* = 72, 53 women, age range: 18–38 years, *M* = 22.3, *SD* = 3.9). Participants who dropped out prior to the last TSST session (*n* = 3) were included in statistical analyses. STRAIN data from one (female) participant were lost due to computer failure.

At the beginning of the presession, participants provided written informed consent, in which they were explicitly informed about their right to withdraw from the study at any time without negative consequences. Upon completion, participants received €15 per appointment as remuneration (€60 in total for full participation). Ethical approval was granted by the Ethics Committee of the University of Siegen (ER_45/2021). Before any data collection took place, the study design and hypotheses were prospectively pre-registered, including an a priori power analysis (https://osf.io/5br2u). Based on a conservative estimate of *r* = .3 for the expected association between endocrine reactivity/habituation and HCC, a target sample size was initially set at *N* = 92 to achieve 90 % power (*α* =.05, two-tailed). By the scheduled end of the study, 82 participants had been recruited after exclusions, resulting in a final sample of *N* = 72 after additional dropouts. Although this number is slightly below the initial target, sensitivity analysis showed that it still provides sufficient power (80 %) to detect effects of the hypothesized magnitude.

### 2.2. Procedure

The study protocol consisted of a total of four standardized sessions: One presession and three consecutive TSST appointments, each scheduled one week apart in the afternoon (between 13:00 and 17:00). During the presession, participants provided written informed consent, and criteria for inclusion were ascertained (in addition to a previous telephone screening interview). Afterward, they completed the STRAIN. Then, a hair sample for HCC analysis was collected (see 2.3.1 for a detailed description). In each of the subsequent sessions, the TSST (Kirschbaum et al., 1993) was administered. Following a resting period of 15–20 min, the TSST consisted of four main phases, each lasting 5 min: instruction, anticipation, free speech, and arithmetic task.

**Fig. 1.**
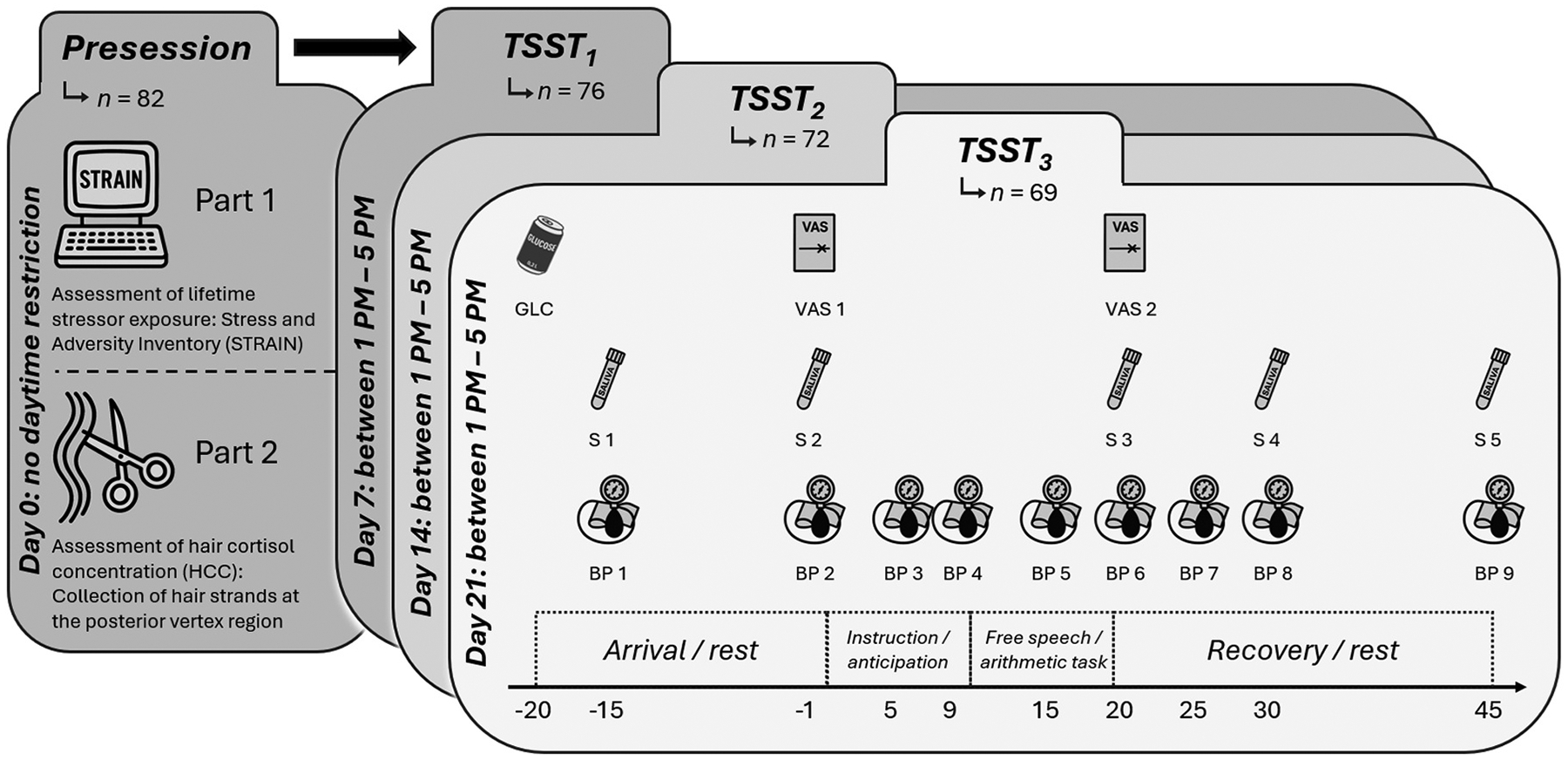
Overview of the study schedule across the presession and three subsequent TSST sessions. Icons above the timeline indicate administration of a glucose drink and measurement timing for a) visual analogue scales (VAS1–2), b) saliva samples (S1–5), and c) blood pressure and heart rate (BP1–9). Abbreviations: TSST: Trier Social Stress Test; STRAIN: Stress and Adversity Inventory; HCC: Hair cortisol concentration; GLC: Glucose; VAS: Visual analogue scale; S: Saliva sample; BP: Blood pressure and heart rate.

Upon arrival at the lab on each TSST session, participants consumed a glucose drink (200 ml, 24 g glucose, cassis juice) and afterwards rinsed their mouth with clear water, in order to control for interindividual variability in baseline blood-glucose levels and to ensure reliable activation of the HPA axis, following current methodological recommendations for standardized TSST implementation (Labuschagne et al., 2019). The TSST consisted of a standardized instruction and anticipation phase, followed by a 5-minute mock job interview and a 5-minute mental arithmetic task performed in front of an evaluative panel. Task parameters (e.g., speech topic, starting number for arithmetic) were counterbalanced across sessions to minimize habituation and sequence effects (Kirschbaum et al., 1993; Labuschagne et al., 2019). After the TSST, the session ended with a 25-minute recovery phase. Throughout the sessions, endocrine, cardiovascular, and subjective stress measures were repeatedly collected (see Fig. 1 for timing). Consistent with standard TSST procedures, participants were informed that their performance would be evaluated and recorded to ensure the presence of social-evaluative threat; all participants were fully debriefed after their final session. As part of the psychosocial stress induction, participants were told that their speech and behavior would be audio- and video-recorded, though no actual recording took place. This deception was upheld across all three TSST sessions until the final debriefing, which occurred immediately after the third TSST session. Participants who discontinued their participation before completion of all sessions were debriefed upon dropout.

### 2.3. Material & measures

#### 2.3.1. Hair cortisol (HCC)

During the presession, hair strands were collected from the posterior vertex region, approximately 1–2 cm above the lambda landmark on the scalp (Huthsteiner et al., 2025). Strands were cut as close to the scalp as possible, with the proximal hair segment used for subsequent endocrine analysis. Before analysis, no sample was stored for longer than three months, following recent recommendations (Huthsteiner et al., 2025b, a). HCC was quantified using liquid chromatography-mass spectrometry (LC-MS), following a well-established procedure by Gao et al. (Gao et al., 2013). To limit the influence of outliers, HCC values (pg/mg) that were more than 1.5 times the interquartile range above or below the median were ‘winsorized’, i.e., replaced with non-extreme maxima/minima.

#### 2.3.2. Salivary cortisol

During the three TSST sessions, saliva samples were collected using Salivette^®^ (Sarstedt) at standardized time points (see Fig. 1). After each appointment, samples were immediately stored at minus 20°C until analysis. Salivary cortisol concentrations were determined using chemiluminescence immunoassay with high sensitivity (Dressendörfer et al., 1992). Before assay, samples were thawed and centrifuged (3000 rpm) for 5 min. The assay’s intra- and inter-assay coefficients of variation were both below 8 %, indicating high reliability.

As salivary cortisol data (nmol/l) were strongly positively skewed (as commonly observed), all values were first log-transformed in order to approximate a normal distribution. Cortisol reactivity (per participant/session) was then determined as the difference between the minimum value during the baseline interval (15, −1 min) before stress induction (i.e., immediately before the anticipation phase of the TSST) and the maximum value after stress exposure (+20, +30, +45 min), following recommendations based on pharmacokinetic modeling of acute cortisol responses (Miller et al., 2013).

#### 2.3.3. Cardiovascular measures

Systolic and diastolic blood pressure, as well as heart rate, were measured using an oscillometric blood pressure monitor (Dinamap PRO 300V2; Critikon, Inc.) at standardized time points (see Fig. 1). Cardiovascular stress reactivity (systolic and diastolic blood pressure, heart rate) was calculated as the difference between the minimum pre-stress value (−15, −1 min) and the maximum value measured during the TSST (+5, +9, +15, +20 min).

#### 2.3.4. Acute subjective stress and arousal: visual analogue scales (VAS)

The current state of subjective stress and arousal was assessed using visual analogue scales (VAS; McCormack et al., 1988) administered immediately before and after the TSST (see Fig. 1). For each measure, participants completed two scales, one assessing stress (“How stressed do you feel?”) and one assessing arousal (“How aroused/excited do you feel?”). Each question was presented with a 10 cm horizontal line labeled with “not at all” on the left and “very much” on the right. Participants were asked to indicate their current state by placing one cross on each line. Afterwards, the distance from the left to the right endpoint was measured and entered as a value ranging from 0.0 to 10.0. Changes in subjectively perceived stress and arousal were defined as pre-post differences (−1 vs. +20 min) relative to stress induction.

#### 2.3.5. Cumulative lifetime stressor exposure: stress and adversity inventory (STRAIN)

Cumulative lifetime stressor exposure was assessed using the STRAIN (Slavich and Shields, 2018) – a detailed and well-validated structured self-administered computerized assessment that measures exposure to a wide variety of acute and chronic stressors across early life and adulthood by systematically integrating stressors of various life domains while accounting for their severity (Slavich and Shields, 2018). More specifically, the STRAIN systematically assesses exposure to 55 distinct acute and past stressors across 12 different life domains (e.g., ‘Accidents’, ‘Financial’, ‘Housing’, ‘Marital/Partner’, etc.). For each indicated stressor, participants answer follow-up questions examining frequency, timing, duration, and perceived severity. Therefore, the STRAIN provides a reliable and nuanced cumulative index of lifetime stress exposure that can be dissociated into different age periods (Slavich and Shields, 2018). As individual stressors are assumed to contribute to an overall cumulative record of exposure rather than reflecting homogenous indices of a single underlying construct, psychometric evaluation of the STRAIN has focused on its test–retest reliability (*r* = .86–.90) and various other validity aspects, rather than internal consistency. Validation studies have demonstrated excellent usability, strong temporal stability (*r* = .87 across two years), and robust convergent (*r* = .45–.70), discriminant, and predictive validity (*r* = .22–.33) in relation to mental and physical health outcomes (Cazassa et al., 2019; Kim et al., 2024; Slavich and Shields, 2018; Sturmbauer et al., 2019). In the present study, the global readout *Total stressor exposure severity* (which aggregates the severity of all stressors) was used for the main correlational analyses.

### 2.4. Data analysis

As a manipulation check of successful stress induction, data of all outcome measures (i.e., stress reactivity indices) were analyzed using linear mixed-effects models with random intercepts per participant, indexing baseline stress reactivity on TSST session 1, and a fixed effect of Session (t_1–3_), reflecting the linear change across repeated exposures (i.e., habituation or sensitization). Significant effects of Session were followed by pairwise comparisons between t_1_ and t_2_ as well as t_2_ and t_3_ (with *p*-values adjusted for multiple testing by means of the Bonferroni method). Furthermore, to obtain unbiased estimates of cortisol reactivity and habituation per individual, random intercepts and random slopes for Session (i.e., linear trends) were estimated and extracted from individual model fits. Models were fitted based on restricted maximum likelihood estimation, using the *lme4* package available in *R* (Bates et al., 2015). Significance of coefficients was assessed by *t*-tests with Satterthwaite approximation.

Because of the heavily skewed distribution of both raw HCC values and stress-related self-report indices, associations with HCC levels (H1 & H2) were first assessed by means of Spearman rank correlations based on untransformed raw data. In the second step, moderation analyses (H3) were performed by means of multiple linear regression. To this end, all variables were approximated to a normal distribution using log-transformation (HCC) or square-root transformation (lifetime stress), respectively (wherever appropriate). Predictors were centered. Separate models with HCC as dependent variable and (1) lifetime stress, cortisol reactivity, and their interaction (H3a) as well as (2) lifetime stress, cortisol habituation, and their interaction (H3b) as predictors were computed. For evaluating H3a and H3b (respectively), these models were compared to an incrementally reduced model without the respective interaction term. Indices of goodness of fit (Akaike Information Criterion, AIC) for these comparisons are given. For ease of interpretation, standardized regression coefficients of the final model are reported. In an additional exploratory analysis, lifetime stressor exposure was separated into early-life and adulthood to examine potential timing effects and their interactions with cortisol reactivity. For each analysis, the threshold of significance was set to α = .05. Furthermore, statistical results at *p* < .10 are discussed as trends.

To make sure that drop-out of participants did not bias the outcome of the study, Little’s MCAR test (Little, 1988) was performed, including the variables age, sex, stress reactivity, HCC, and total stressor exposure severity. The test was not significant (χ^2^(15) = 16.89, *p* = .33), indicating that data were missing completely at random.

## 3. Results

### 3.1. Stress reactivity and habituation (manipulation check)

**Fig. 2.**
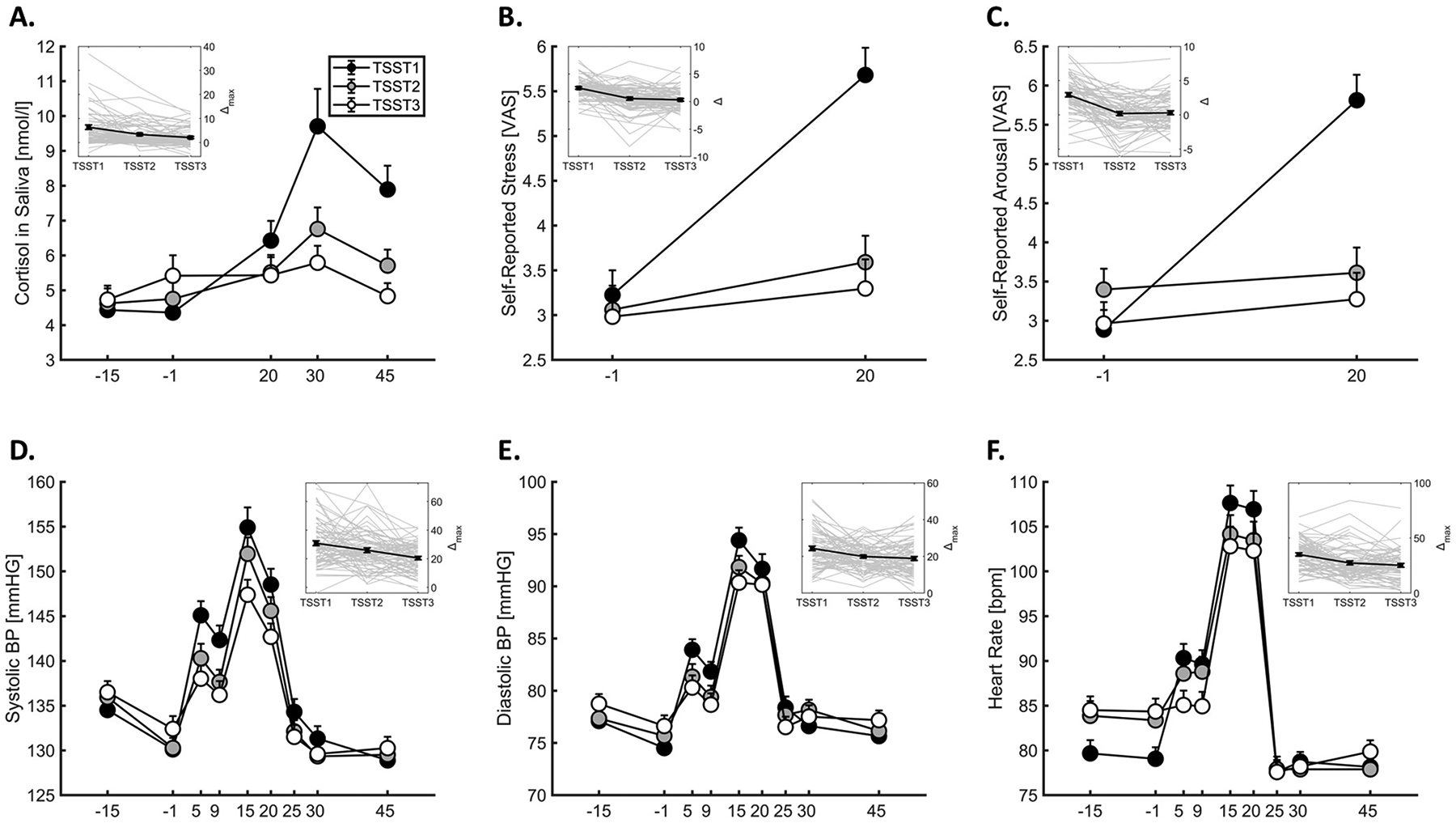
Changes in stress indices during TSST sessions 1, 2, and 3 (at intervals of one week): A. salivary cortisol, B. self-reported stress, C. self-reported arousal, D. systolic blood pressure, E. diastolic blood pressure, and F. heart rate. X-axis: time (min) relative to the start of the TSST (anticipation phase). Embedded plots show trajectories of stress reactivity (maximum pre-post change) across repeated exposures (mean and individual data). *Note*. BP: blood pressure; VAS: visual analogue scale. Error bars represent *SEM*.

For demographic characteristics of the study sample, see Table 1. Following the first laboratory-based stress exposure (*TSST*_*1*_), there were significant increases (Δ_max_) in salivary cortisol (*b*_*0*_ = 0.82 [log(nmol/l)], *SE* = 0.07, *t*[147.0] = 11.97, *p* < .001), systolic blood pressure (*b*_*0*_ = 30.7 [mmHG], *SE* = 1.43, *t*[170.2] = 21.41, *p* < .001), diastolic blood pressure (*b*_*0*_ = 23.1 [mmHG], *SE* = 0.96, *t*[175.7] = 24.07, *p* < .001), heart rate (*b*_*0*_ = 34.1 [bpm], *SE* = 1.60, *t*[123.7] = 21.28, *p* < .001), self-reported stress (*b*_*0*_ = 2.18 [VAS], *SE* = 0.23, *t*[171.5] = 9.39, *p* < .001) and self-reported arousal (*b*_*0*_ = 2.45 [VAS], *SE* = 0.29, *t*[152.1] = 8.37, *p* < .001). Fig. 2 shows the mean raw values of all stress indices across sampling points and sessions. Significant negative linear trends across repeated exposures (reflecting habituation of stress reactivity) were found for all outcome measures (salivary cortisol: *b* = −0.23, *SE* = 0.04, *t*[140.9] = −5.57, *p* < .001; systolic blood pressure: *b* = −5.0, *SE* = 0.96, *t*[142.2] = −5.27, *p* < .001; diastolic blood pressure: *b* = −2.3, *SE* = 0.65, *t*[141.9] = −3.58, *p* < .001; heart rate: *b* = −4.72, *SE* = 0.85, *t*[140.1] = −5.57, *p* < .001; self-reported stress: *b* = −1.09, *SE* = 0.16, *t*[142.3] = −6.95, *p* < .001; self-reported arousal: *b* = −1.30, *SE* = 0.18, *t*[140.5] = −7.15, *p* < .001). Follow-up analyses further showed that reactivity scores differed significantly between *TSST*_*1*_ and *TSST*_*2*_ for all measures (all *p*s < .03; Bonferroni-corrected), whereas differences between *TSST*_*2*_ and *TSST*_*3*_ were only significant for cortisol (*p* = .024) and systolic blood pressure (*p* = .011), yet not for diastolic blood pressure (*p* = .89), heart rate (*p* = .35), self-reported stress (*p* > .9) and arousal (*p* > .9). The proportion of participants showing a cortisol increase ≥ 1.5 nmol/L declined from 72.4 % at TSST1–52.8 % at TSST2 and 37.7 % at TSST3, indicating progressive habituation of the HPA-axis response. Moreover, cortisol peaks occurred predominantly at 30 min after stress onset during TSST1 (55.3 %), but shifted toward earlier peaks at 20 min (TSST1: 13.2 %, TSST2: 34.7 %, TSST3: 39.1 %; see Supplement for details). Analyses examining the influence of testing time (13:00–17:00) on baseline cortisol, peak levels, and cortisol reactivity (Δ) revealed no evidence of significant time-of-day effects (all *p*s > .4). Notably, there was also no indication of any systematic differences in baseline levels between sessions for any reactivity measure (all *F*s < 1, *p*s > .5), except for heart rate, which showed significantly enhanced pre-stress levels on session 2 and 3 (*F*[2211] = 4.24, *p* = .015)

### 3.2. Association of hair cortisol with lifetime stress exposure (hypothesis 1)

**Fig. 3.**
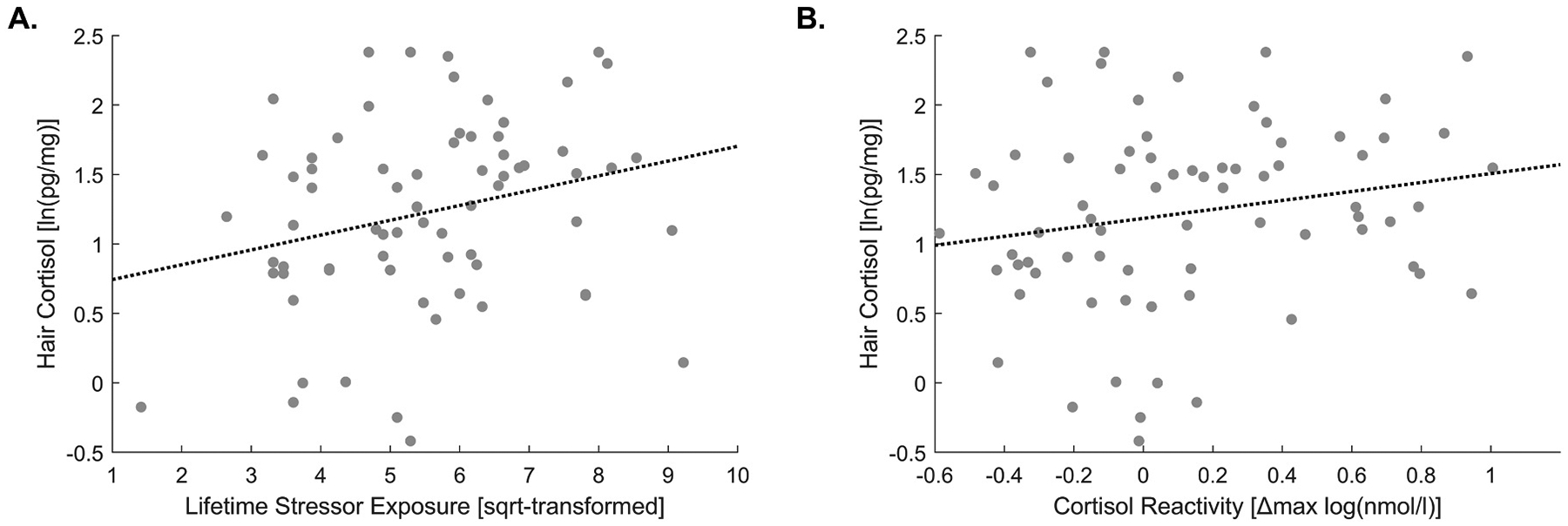
Associations of HCC with (A) lifetime stressor exposure and (B) cortisol stress reactivity (during the first TSST).

Consistent with our pre-registered hypothesis (H1), greater lifetime stressor exposure (*M* = 33.2, *SD* = 18.7) was related to higher HCC levels (*M* = 1.22 [log(pg/mg)], *SD* = 0.66): *ρ* = .27, *p* = .021. See Fig. 3 (A) for illustration.

### 3.3. Association of hair cortisol with stress reactivity and habituation (hypothesis 2)

The correlation of HCC with individual cortisol reactivity, as derived from the first TSST (*M* = 0.133 [Δ_max_log(nmol/l)], *SD* = 0.409) (H2a), approached significance: *ρ* = .21, *p* = .078 (see Fig. 3B). Contrary to expectations (H2b), there was no significant association between HCC and the habituation of cortisol reactivity (i.e., individual slopes across sessions; *M* = −0.11, *SD* = 0.18): *ρ* = −.15, *p* = .205 (see Fig. 3B). Notably, cortisol reactivity at t_1_ was inversely correlated with habituation (*r* = −.63, *p* < .001).

### 3.4. Moderation by stress reactivity and habituation (hypothesis 3)

As evident from multiple regression analyses (see Table 2), neither cortisol reactivity (H3a, model A1) nor its habituation (H3b, model B1) moderated the association between lifetime stressor exposure and HCC (all interaction terms: *p*s > .49). However, both cortisol reactivity (β = 0.24; *SE* = 0.11, *t*[68] = 2.11, *p* = .039) and lifetime stressor exposure (β = 0.30; *SE* = 0.11, *t*[68] = 2.61, *p* = .011) emerged as significant predictors of HCC (model A0; adjusted multiple *R*^2^ =.101; see Table 2). Notably, these associations also proved robust when tested within a single regression model (including both cortisol reactivity and habituation) as well as when accounting for the potential influence of variation in baseline cortisol levels (see Supplement for details).

In addition, we checked for the influence of potential covariates (i.e., BMI, age, hair treatment, hair-washing frequency, and sex; Stalder et al., 2017). None of these variables were significantly correlated with HCC in our sample (*p*s > .3), except for sex (*r* = .28, *p* = .020). Notably, when controlling for all covariates simultaneously, the association with lifetime stressor exposure remained significant (*p* = .007), while the effect of cortisol reactivity did not (*p* = .113).

As suggested during peer review, we additionally tested whether the main findings of our study were robust after exclusion of participants with chemically treated hair (remaining sample: *n* = 52): There was still a significant, numerically even stronger HCC/lifetime stress correlation (*ρ* =.33, *p* = .020), but no significant association with cortisol reactivity (*ρ* =.15, *p* = .286; habituation: *ρ* = −.21, *p* = .149). Also, the overall pattern of associations remained largely unchanged when controlling for baseline variation in cortisol (across participants and sessions) using covariate-adjusted peak levels as an alternative reactivity metric. See Supplement for detailed results of all additional (covariate) analyses.

### 3.5. Role of early-life vs. adult-life stress (exploratory analyses)

**Fig. 4.**
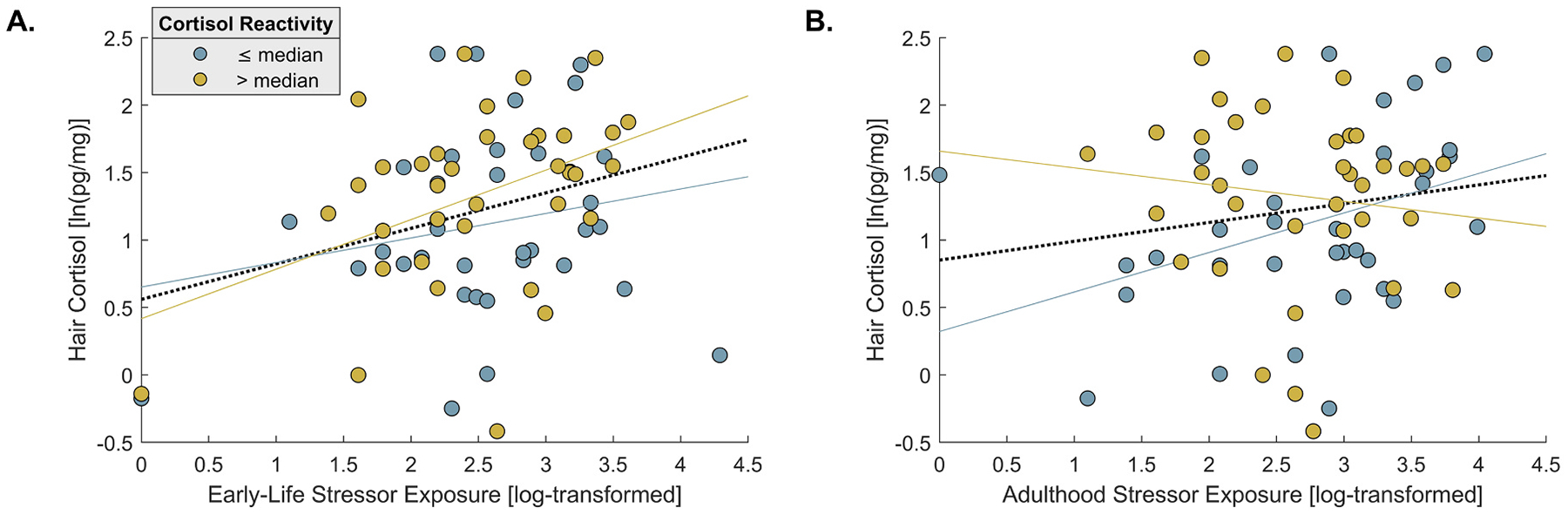
Associations of HCC with age-related dimensions of lifetime stressor exposure, shown as a function of cortisol reactivity (median split): A. early-life stressor exposure, B. adulthood stressor exposure.

To test for potential differences in the contribution of early-life vs. adulthood stressor exposure in relation to HCC, including their potential interplay with cortisol reactivity, we performed an additional moderation analysis: HCC was regressed on STRAIN-derived scores of both early-life and adulthood life stressor exposure, as well as their interactions with cortisol reactivity. This analysis revealed a significant association between HCC and early-life adversity (β = 0.26; *SE* = 0.11, *t*[66] = 2.26, *p* = .028). The interaction of early-life stressor exposure with cortisol reactivity was not significant (*p* = .215). For adulthood stressor exposure, the main effect was non-significant (β = 0.13; *SE* = 0.11, *t*[66] = 1.14, *p* = .261), but a significant interaction with cortisol reactivity emerged (β = −0.26; *SE* = 0.11, *t*[66] = −2.30, *p* = .025). The main effect of cortisol reactivity was at trend level (β = 0.20; *SE* = 0.11, *t*[66] = 1.77, *p* = .081; adulthood life stressor exposure: β = 0.13; *SE* = 0.11, *t*[66] = 1.14, *p* = .261). Overall, this model explained 16.6 % of variance in HCC levels (adjusted *R*^*2*^). Including sex led to a marginal increase in explained variance ($R_{\text{adjusted}}^{2}=.198$; *F*[1,65] = 3.62, *p* = .062), but did not change the significance of the interaction terms or the pattern of results. The same pattern was also observed when using an alternative metric of cortisol reactivity, covariate-adjusted for baseline levels (see Supplement). As illustrated in Fig. 4, our data suggest a consistent link between early-life adversity and HCC. Conversely (and unexpectedly), the association between adulthood stressor exposure and HCC weakened with rising levels of cortisol reactivity.

## 4. Discussion

The present study set out to investigate how neuroendocrine reactivity to a psychosocial stressor, as well as stress-response habituation, relates to HCC and its putative link to lifetime stressor exposure. As expected, the first exposure to the TSST elicited substantial increases in all physiological and self-report indices of stress reactivity, whereas subsequent habituation was evident from negative linear trends across repeated TSST sessions (observed for all measures). Furthermore, consistent with our pre-registered hypotheses, HCC was associated with both lifetime stressors (H1) and cortisol reactivity (H2a). In contrast, no association emerged between HCC and habituation of cortisol reactivity (H2b), and the predicted moderation effect of neither stress reactivity (H3a) nor its habituation (H3b) on the HCC-stress link was supported by the data. However, exploratory analyses targeting distinct contributions of early-life vs. adulthood stressor exposure suggested a partial modulatory role of cortisol reactivity for the association between stressors occurring in adulthood and HCC, whereas early-life adversity was consistently linked to elevated HCC.

Supporting our first hypothesis (H1), we found that individuals reporting higher levels of cumulative life stress also exhibited modestly elevated HCC. Beyond prior evidence for group-level associations with varying facets of self-reported stress (Planert et al., 2023; Stalder et al., 2017), this finding corroborates the potential value of HCC as an index of chronic stressor exposure, based on a more detailed, behaviorally anchored measure of stress across the life span. Previous meta-analytic findings revealed elevated HCC in high stress groups, particularly with ongoing stressor exposure, but generally failed to reveal consistent associations with questionnaire-based assessments of chronic perceived stress (Stalder et al., 2017). HCC thus presumably reflects long-term cortisol output (under stressed or unstressed conditions) but may not fully represent the psychological experience of stress. Accordingly, while our data support a link between lifetime stressor exposure and HCC, this relationship likely captures physiological imprinting of cumulative stressor exposure rather than chronic stress itself. Also, despite the observed relationship, the use of the STRAIN did not substantially increase the strength of the association, contrary to expectations, despite providing a more comprehensive framework for stress assessment. One possible explanation may be the fact that we employed the STRAIN in a non-clinical, high-functional sample, where overall lifetime stressor exposure tends to be lower and less pathological (Slavich and Shields, 2018). Moreover, consistent with prior research, small-to-medium effect sizes suggest that HCC is not a homogenous index of chronic stress exposure, but rather an integrative marker influenced by various other processes, including individual and/or contextual factors (Russell et al., 2012).

One such factor may be stress reactivity, with which HCC was marginally associated in our study (consistent with expectations, H2a), indicating that individuals with higher HCC had a slightly increased response to acute, psychosocial stress. Presumably, the intensity of acute cortisol responses may accumulate over time, resulting in higher levels of HCC. This interpretation aligns with prior research that provided preliminary evidence for a direct link between the magnitude of the cortisol response to the TSST and HCC (Steudte-Schmiedgen et al., 2015; but see Sandner et al., 2020). For example, Steudte-Schmiedgen et al. (2015) found a marginal correlation between acute stress reactivity and HCC. By contrast, other research (Sandner et al., 2020) suggested that elevated HCC is associated with lower acute stress responses, which could indicate a history of chronic activation or blunted responsivity of the HPA axis. Regarding the latter, at higher levels of cumulative stressor exposure, the regulatory efficiency of the HPA axis may deteriorate, resulting in attenuated cortisol responses despite elevated basal output (Fries et al., 2005; McEwen, 2007). Consequently, the association between stress reactivity and long-term cortisol accumulation is unlikely to be strictly linear (Miller et al., 2007). Heterogeneity in findings highlights the inherent complexity and current lack of understanding of HCC as a marker of individual stress sensitivity. Some inconsistencies may be partly explained by differences in sample characteristics, study design, or analytical strategy. Steudte-Schmiedgen et al. (2015) examined a military cohort including individuals with and without PTSD, whereas Sandner et al. (2020) investigated a small, non-clinical student sample. In addition, the two studies used distinct stress paradigms (TSST versus ScanSTRESS-C task) and differed in their temporal design, with Steudte-Schmiedgen et al. relating baseline HCC to prospective outcomes, while Sandner et al. assessed cross-sectional associations within a single session. Such methodological variation may contribute to discrepancies in the observed direction and strength of association between HCC and acute stress reactivity. In our study, cortisol reactivity also did not reliably correlate with HCC when further covariates, particularly hair treatment, were taken into account. The conflicting pattern of results also underscores the necessity to investigate its relation to the temporal dynamic of stress reactivity, which presumably reflects an individual’s ability to adapt to repeated exposure to the same type of stressor.

While decreases in response magnitude from the first to the second TSST exposure were found for all stress reactivity indices in our study, cortisol responses continued to habituate into the third session, as did changes in systolic blood pressure (whereas other indices of cardiovascular reactivity did not, similar to prior findings indicating reduced habituation of autonomic stress responses (Finke et al., 2022; Schommer et al., 2003). Most importantly, no association was found between HCC and the habituation of cortisol reactivity, unlike predicted by our hypothesis H2b. At first glance, this finding suggests that HCC may not directly reflect successful long-term adaptation to stressors. The endocrine mechanisms underlying cortisol habituation are complex and operate on different temporal scales. Rapid habituation (within minutes or hours) depends on efficient negative feedback regulation through glucocorticoid receptors, which serve to terminate acute HPA-axis activation (Herman et al., 2016; Kloet et al., 2005). However, such receptor-based feedback adjustments are unlikely to change fundamentally across a few laboratory stress exposures and, therefore, also unlikely to serve as an explanation for adaptation spanning over several weeks or longer (McEwen, 1998). Beyond endocrine processes, adaptation to stressors is also shaped by cognitive and affective factors (Gianferante et al., 2014; Schommer et al., 2003; Zoccola and Dickerson, 2012) and, in this context, stress habituation can be better understood as a dynamic process resulting from contextual learning, emotional (re-)appraisal, and situational predictability (Grissom and Bhatnagar, 2009). Over longer timescales, more persistent modifications in HPA-axis function may occur, such as receptor (up/down) regulation or altered set points following highly intense stressor exposure, especially at an early period in life (Meaney and Szyf, 2005; Miller et al., 2007). Taken together, the accumulation of HCC may lack the temporal resolution required to capture this broad range of nuanced regulatory mechanisms. Interestingly, however, habituation and initial reactivity were strongly negatively correlated in the present sample, mirroring findings from Wüst et al. (2005) and Kudielka et al. (2006), who reported that individuals with low cortisol responses were also more likely to yield weak habituation. This supports the idea that initial responsivity and flexibility in stress adaptation are meaningfully linked, consistent with the concept of allostasis and allostatic load, in which the short-term stress response is protective, while the inability to adapt to repeated stressors may result in dysregulation of the endocrine system (Kudielka et al., 2009; McEwen, 1998b). Given this premise, the lack of association between habituation and HCC may indicate that HCC is more sensitive to long-term strain than to adaptability to repeated stress. Therefore, conceptualizing HCC as the final output of short-term, stress-related fluctuations in cortisol is probably too simplistic. Rather, different neuroendocrine measures with varying temporal resolutions likely capture distinct aspects of stress physiology (and may differentially predict specific physical and mental health outcomes).

Contrary to our third hypothesis, we found no evidence of either stress reactivity (H3a) or its habituation (H3b) moderating the association between lifetime stressor exposure and HCC. The rationale behind the hypothesis was that elevated stress sensitivity or impaired stress adaptation might lead to a larger endocrine imprint of chronic stress in HCC, potentially explaining the inconsistent pattern of previous reports. However, lifetime stressor exposure and stress reactivity were separately associated with HCC, suggesting that this association may be more additive than interactive, at least in the present healthy sample. At the same time, the absence of the predicted moderation does not imply that the underlying regulatory mechanisms are irrelevant. For instance, the results contrast with prior research suggesting that long-term stress-related outcomes are directly shaped by processes of stress adaptation, such as stress reactivity or habituation (Kudielka et al., 2009; Wüst et al., 2005). Moreover, inter-individual variability in habituation has been linked to psychological traits such as self-esteem (Abouserie, 1994), locus of control (Kirschbaum et al., 1995), and post-stress rumination (Gianferante et al., 2014), which may modulate how effectively individuals biologically embed stress over time. From this point of view, the absence of moderation effects in our study could also reflect limited variability in these traits.

In addition to the pre-registered analyses concerning the main hypotheses, we conducted an additional analysis disentangling lifetime stressor exposure into early-life vs. adulthood stressor exposure. Interestingly, this analysis revealed that while experiencing early-life adversity can lead to elevated HCC, stressors occurring during adulthood showed a diminishing association with HCC as individual cortisol reactivity increased. The link between early-life adversity and HCC is consistent with prior research suggesting that stress in sensitive, developmental periods exerts lasting effects on HPA-axis activity, which might be reflected in accumulated HCC (Hengesch et al., 2018; Marsland et al., 2024). Consistent with set point models of HPA-axis regulation, early-life stress may induce long-term, epigenetically caused alterations in glucocorticoid receptor expression, which may either heighten or dampen negative feedback sensitivity (Miller et al., 2007; Meaney and Szyf, 2005). Dampened feedback sensitivity likely leads to greater basal cortisol secretion (Fries et al., 2009; Gunnar and Quevedo, 2007), thereby potentially increasing cumulative cortisol deposition in hair. However, at present, this remains speculation, and meta-analytic evidence has so far not revealed stronger associations for early versus more recent stressor exposure (Stalder et al., 2017). Importantly, early adversity may also be related to neuroplasticity, i.e., structural and functional changes of the brain during early development, in regions that strongly impact the HPA-axis, such as the amygdala and the hippocampus (Dannlowski et al., 2012). This may constitute further pathways contributing to the early adversity-HCC link.

In contrast, for stressor exposure during adulthood, the association with HCC was weaker in individuals with higher cortisol reactivity in our study. Though rather unexpected, this pattern aligns with prior evidence for reduced phasic cortisol responses under conditions of greater chronic HPA-axis activation (Kudielka et al., 2009; Voellmin et al., 2015), potentially reflecting ceiling effects in HPA-axis drive: Spikes in acute stress reactivity resulting from low basal cortisol may contribute less to HCC than tonic cortisol secretion in individuals who thus show limited capacity for further cortisol increase in the face of an acute stressor (Kudielka et al., 2009; Schlotz et al., 2011). This may explain weaker overall associations between adulthood stressor exposure and HCC, even though, unlike early-life adversity, adulthood stressor exposure may not entail fundamental changes to HPA set points, which are assumed to remain largely intact at this developmental stage (Lupien et al., 2009; Tarullo and Gunnar, 2006). Combined, these mechanisms might account for the fact that early adversity was associated with higher HCC irrespective of current reactivity, whereas stress experiences later in life appear to produce more variable endocrine response profiles.

### 4.1. Limitations & future directions

While the current study provided novel insight into the relationship between HCC, lifetime stress exposure, stress reactivity, and its habituation, several limitations need to be acknowledged. First, due to the homogeneity of the predominantly female, young, and non-clinical sample, variability in stress exposure may have been limited. Despite some advantages in terms of minimizing the influence of confounding variables, the present results should therefore be interpreted with caution and may not generalize to other (e.g., older or clinical) populations. Given the comparably small proportion of male participants, it was not feasible to reliably assess potential sex effects. Prior research has, for example, documented systematic sex differences in the activity of the HPA axis and cortisol reactivity (Kajantie and Phillips, 2006; Kirschbaum et al., 1999). Such sex specific hormonal profiles may alter the pattern of psychoneuroendocrine covariance to a certain extent, thus limiting the external validity of our findings. Also, as no structured clinical interview was administered, subclinical psychological symptoms cannot be fully ruled out, and future research should incorporate standardized diagnostic assessments to ensure more precise characterization of mental health status. Future studies could replicate our study in clinical and more diverse samples. Furthermore, the generalizability of the present results is also constrained by our decision to include only women using oral contraceptives. While this procedure ensured standardization of hormonal influences on cortisol secretion, especially given that the study took four weeks to complete, future studies could try other potential solutions, such as a shorter assessment period, e.g., administration of multiple TSSTs within a single menstrual phase. Using the TSST, a well-validated and established stress protocol linked to pronounced cortisol responses (Kirschbaum et al., 1993), allows for meaningful comparison with other studies. However, it is unclear whether alternative methods of stress induction, relying on other stressors than social-evaluative threat, might lead to divergent results (Finke et al., 2018, 2021). Also, robustness checks that included demographic and hair-related covariates produced the same overall conclusion for lifetime stressor exposure but reduced the contribution of cortisol reactivity. Moreover, because baseline saliva was collected shortly after glucose intake, even though participants rinsed their mouth with water, a minor influence of the drink on baseline cortisol cannot be ruled out. Beyond these sample- or design-specific considerations, our exploratory findings suggest that a promising avenue for future research would be to focus on how developmental timing, chronicity, and subjective appraisal affect the accumulation of HCC.

## 5. Conclusion

The present study was the first to examine how lifetime stressor exposure, stress reactivity, and habituation are jointly related to HCC. Our aim was twofold: (1) providing an in-depth assessment of lifetime stressor exposure (via the STRAIN) as well as its association with HCC, and (2) examining the potential modulatory role of stress reactivity and its habituation. To conclude, our findings underscore the potential of HCC to index not only long-term stress exposure but also individual variability in stress responsivity. Together with the observed link between early-life adversity and HCC, both the extent of reacting to acute stressors and experiencing stress during crucial developmental periods may leave a lasting endocrine imprint. Furthermore, the absent association with stress habituation points towards HCC being less sensitive to short-term adjustments in the stress response. Rather than serving as a homogenous readout of accumulated lifetime stress, HCC may also capture information on how stress is physiologically processed over time. Future research may further disentangle the underlying components to enhance our understanding of the predictive value of HCC as a biomarker of stress and related health risks.

## Supplementary Material

Supplementary Material

## Figures and Tables

**Table 1:** Demographic characteristics of the study sample (*N* = 72).

Variable	*M*	*SD*
Age (years)	22.5	4.1
Gender (female)	73.6 %	
BMI (kg/m^2)	23	3.4
HCC (pg/mg)	4.3	3.4
Hair treatment	30.6 %	
Hair washing frequency (per week)	3.8	1.7

*Note*. BMI, body-mass-index; HCC, hair cortisol concentration.

**Table 2:** Goodness of fit (AIC) of regression models evaluating moderation hypotheses (H3a: A1 vs. A0; H3b: B1 vs. B0).

Model (predictors)	$R_{\text{adjusted}}^{2}$	df_residuals_	df_predictors_	AAIC	*F*	*p*	
**A1**. Cortisol reactivity*Lifetime stress, Cortisol reactivity, Lifetime stress, intercept	.094	67	3	1.5	0.47	.497	A1 vs. A0
**A0**. Cortisol reactivity, Lifetime stress, intercept	.101	68	2	−4.1	4.91	.010	A0 vs. C
**B1**. Cortisol habituation*Lifetime stress, Cortisol habituation, Lifetime stress, intercept	.053	67	3	1.8	0.20	.655	B1 vs. B0
**B0**. Cortisol habituation, Lifetime stress, intercept	.065	68	2	−2.8	3.41	.039	B0 vs. C
**C**. Intercept	0	70	0				

*Note*. ΔAIC: Change in Akaike Information Criterion (model comparison). Lifetime stress represents the cumulative lifetime stressor exposure as measured by the Stress and Adversity Inventory.

## Appendix A. Supporting information

Supplementary data associated with this article can be found in the online version at doi:10.1016/j.psyneuen.2025.107715.
