# Supplementary material for: Linking hair cortisol and life stress: The role of stress reactivity and habituation
Source: Psychoneuroendocrinology. Author manuscript; Available in PMC 2026 Mar 18. (PMC12998516; doi:10.1016/j.psyneuen.2025.107715)
Supplement: Supplementary Material [file NIHMS2155082-supplement-Supplementary_Material.pdf]

Supplementary Material

Linking hair cortisol and life stress:
The role of stress reactivity and habituation

Planert, Stalder, Huthsteiner, Slavich, Klucken, & Finke, 2025, *Psychoneuroendocrinology*

A) Cortisol response patterns

The distribution of cortisol reactivity profiles (sampling times following stress exposure corresponding to individual peak cortisol levels) across all three TSST sessions, along with the proportion of 'cortisol responders,' is given in Table S1.

Table S1. Proportion of cortisol reactivity profiles per session

Session	Peak at 20 min (%)	Peak at 30 min (%)	Peak at 45 min (%)	Responder (%) ($\Delta_{max} \geq 1.5$ nmol/L)
TSST1	13.16	55.26	31.58	72.40
TSST2	34.72	43.06	22.22	52.80
TSST3	39.13	44.93	15.94	37.70

B) Exploratory covariate analyses

Detailed regression analyses including all theoretically relevant variables (linked to HCC levels in previous research) as covariates are reported below. Please note that, due to the limited sample size, these analyses may be somewhat underpowered and should therefore be interpreted with caution. Table S2 provides the results of both main analyses (a: H3a, b: H3b) when controlling for the potential influence of these covariates.

Table S2. Regression (standardized coefficients) of hair cortisol concentration (HCC) on stress-related variables, including HCC-related covariates

Model term	*β*	SE	*t*	*p*	*Adjusted R^2*
(a)					.120*
Gender (male)	0.25	0.14	1.80	.077 °	
Age (years)	-0.18	0.13	-1.36	.178	
Body Mass Index	0.19	0.12	1.61	.113	
Hair treatment	0.01	0.12	0.04	.968	
Hair washing frequency	0.07	0.12	0.62	.536	
Cortisol reactivity (CR)	0.20	0.12	1.61	.113	
Lifetime stress (LS)	0.33	0.12	2.77	.007**	
CR*LS	-0.12	0.11	-1.08	.286	
(b)					.073
Gender (male)	0.30	0.15	1.97	.053 °	
Age (years)	-0.14	0.13	-1.06	.292	
Body Mass Index	0.16	0.12	1.33	.188	
Hair treatment	0.02	0.13	0.16	.871	
Hair washing frequency	0.08	0.12	0.66	.512	
Cortisol habituation (CH)	-0.02	0.13	-0.16	.877	
Lifetime stress (LS)	0.30	0.12	2.48	.016*	
CH*LS	0.04	0.12	0.30	.763	

Note. $N = 71$. SE, standard error; levels of significance: ° $p < .10$, * $p < .05$, ** $p < .01$

C) Alternative analysis of cortisol reactivity: peak scores of log-transformed saliva cortisol (nmol/l), covariate-adjusted for baseline levels

To provide an additional robustness check, we examined whether variation in baseline cortisol (across participants and sessions) affected cortisol reactivity and, in turn, its association with HCC levels. Instead of calculating change scores (Δ_{max}) by subtracting pre-stress baseline levels, a linear mixed-effects model was fitted to predict individual post-stressor peak levels from (mean-centered) baseline cortisol and session (fixed effects), with random intercepts and random slopes for session by participant. Participant-specific intercepts and slopes were extracted, reflecting each individual's peak cortisol response at the first session and linear trends across sessions, respectively, adjusted for differences in baseline cortisol. These parameters were used as alternative indices of cortisol reactivity and habituation in the following correlational analyses.

C1. Habituation of cortisol reactivity (manipulation check)

Following the first laboratory-based stress exposure ($TSST_1$), post-stressor peak levels in salivary cortisol (b_0 = 2.02 [log(nmol/l)], *SE* = 0.07) significantly declined across repeated exposures (*b* = -0.20, *SE* = 0.04, *t*[140.1] = -5.61, $p < .001$). There was also a significant positive association with baseline cortisol (*b* = 0.50, *SE* = 0.07, *t*[209.9] = 7.26, $p < .001$).

C2. Association of hair cortisol with stress reactivity and habituation (hypothesis 2)

The correlation of HCC with individual cortisol reactivity, as derived from the first TSST, approached significance: $\rho = .23$, $p = .054$. There was no significant association between HCC and the habituation of cortisol reactivity ($\rho = -.12$, $p = .337$), which was inversely correlated with cortisol reactivity at t_1 ($r = -.63$, $p < .001$).

C3. Moderation by stress reactivity and habituation (hypothesis 3)

Multiple regression analyses (see Table S3) revealed that neither cortisol reactivity (model A1) nor its habituation (model B1) moderated the association between lifetime stressor exposure and HCC (all interaction terms: *p*s > .9), when additionally controlling for baseline cortisol. However, largely consistent with the main analysis, both lifetime stressor exposure (β = 0.32; *SE* = 0.12, *t*[67] = 2.67, *p* = .009) and (marginally) cortisol reactivity (β = 0.21; *SE* = 0.11, *t*[67] = 1.86, *p* = .068) emerged as predictors of HCC (model A0; adjusted multiple R^2 = .090; see Table S3). The influence of baseline cortisol was not significant (β = -0.15; *SE* = 0.12, *t*[67] = -1.29, *p* = .203)

Table S3. Goodness of fit (AIC) of regression models evaluating moderation hypotheses (H3a: A1 vs. A0; H3b: B1 vs. B0), controlling for baseline cortisol (baseline-adjusted peak scores of cortisol and baseline cortisol as covariate)

**Model (predictors)**	$R^2_{adjusted}$	$df_{residuals}$	$df_{predictors}$	**ΔAIC**	***F***	***p***	
**A1.** Cortisol reactivity*Lifetime stress, Cortisol reactivity, Lifetime stress, Baseline cortisol (t_1), intercept	.077	66	4	1.9	0.00	.961	A1 vs. A0
**A0.** Cortisol reactivity, Lifetime stress, Baseline cortisol (t_1), intercept	.090	67	3	-2.0	3.32	.025	A0 vs. D
**B1.** Cortisol habituation*Lifetime stress, Cortisol habituation, Lifetime stress, Baseline cortisol (t_1), intercept	.041	66	4	1.8	0.20	.655	B1 vs. B0
**B0.** Cortisol habituation, Lifetime stress, Baseline cortisol (t_1), intercept	.055	67	3	-2.8	2.35	.080	B0 vs. D
**C.** Baseline cortisol (t_1), intercept	-.013	69	1	1.9	0.12	.728	C vs. D
**D.** Intercept	0	70	0				

Note. ΔAIC: Change in Akaike Information Criterion (model comparison). Lifetime stress represents the cumulative lifetime stressor exposure as measured by the Stress and Adversity Inventory.

C4. Role of early-life vs. adult-life stress (exploratory analyses)

Similar to the analysis reported in the main text, the model assessing specific effects of early-life stressor exposure, adulthood stressor exposure, and their interactions with cortisol reactivity (controlling for baseline cortisol) showed no significant main effect of adulthood stressor exposure on HCC ($\beta = 0.17$; $SE = 0.12$, $t[64] = 1.41$, $p = .165$), but a significant interaction with cortisol reactivity ($\beta = -0.28$; $SE = 0.13$, $t[64] = -2.14$, $p = .036$) as well as a simple main effect of early-life stress ($\beta = 0.26$; $SE = 0.12$, $t[64] = 2.26$, $p = .027$), but no significant interaction with cortisol reactivity ($\beta = 0.17$; $SE = 0.11$, $t[64] = 1.52$, $p = .134$). There was also a marginal effect of (baseline-adjusted) cortisol reactivity ($\beta = 0.19$; $SE = 0.11$, $t[64] = 1.67$, $p = .099$), but no (direct) association of HCC with baseline cortisol ($\beta = -0.05$; $SE = 0.12$, $t[64] = -0.43$, $p = .672$). Overall, this model explained 14.1% of variance in HCC levels (adjusted R^2).

C5. Exploratory covariate analyses based on baseline-adjusted peak scores (cortisol reactivity)

When controlling for the influence of baseline cortisol as well as all other (potentially) HCC-related variables as covariates at once, the relationship between HCC and cortisol reactivity was no longer significant ($p = .424$). By contrast, the HCC/lifetime stress association proved robust ($p = .005$). See Table S4 for detailed results of reactivity (H3a) as well as habituation (H3b) models.

Table S4. Regression (standardized coefficients) of hair cortisol concentration (HCC) on stress-related variables, including HCC-related covariates, controlling for baseline cortisol (baseline-adjusted peak scores of cortisol and baseline cortisol as covariate)

**Model term**	***β***	**SE**	***t***	***p***	***Adjusted R^2***
(a)					.103 °
Baseline cortisol (t_1)	-0.18	0.12	-1.49	.141	
Gender (male)	0.29	0.15	2.00	.049*	
Age (years)	-0.13	0.14	-0.99	.325	
Body Mass Index	0.17	0.12	1.41	.165	
Hair treatment	0.01	0.13	0.10	.922	
Hair washing frequency	0.07	0.12	0.54	.592	
Cortisol reactivity (CR)	0.11	0.13	0.80	.424	
Lifetime stress (LS)	0.36	0.12	2.89	.005**	
CR*LS	-0.04	0.10	-0.43	.670	
(b)					.108 °
Baseline cortisol (t_1)	-0.18	0.12	-1.48	.143	
Gender (male)	0.39	0.15	2.58	.012*	
Age (years)	-0.12	0.13	-0.93	.354	
Body Mass Index	0.17	0.12	1.47	.146	
Hair treatment	0.02	0.13	0.19	.847	
Hair washing frequency	0.08	0.12	0.63	.533	
Cortisol habituation (CH)	0.06	0.13	0.44	.664	
Lifetime stress (LS)	0.34	0.12	2.76	.007**	
CH*LS	0.12	0.12	1.05	.299	

Note. $N = 71$. SE, standard error; levels of significance: ° $p < .10$, * $p < .05$, ** $p < .01$
